# Exosome-coated polydatin nanoparticles in the treatment of radiation-induced intestinal damage

**DOI:** 10.18632/aging.204882

**Published:** 2023-07-18

**Authors:** Qiu Chen, Lei Yao, Quanbin Liu, Jun Hou, Xinyu Qiu, Mengyuan Chen, Zhuojun Wu, Duanmin Hu, Fengmei Cui, Tao Yan

**Affiliations:** 1 State Key Laboratory of Radiation Medicine and Protection, School of Radiation Medicine and Protection, Soochow University, Suzhou 215123, China; 2 Collaborative Innovation Center of Radiation Medicine of Jiangsu Higher Education Institutions, Suzhou 215123, China; 3 Rocket Force Specialty Medical Center PLA, Beijing 100088, China; 4 Department of Pathology, Zhongshan Hospital, Fudan University, Shanghai 200032, China; 5 Department of Gastroenterology, The Second Affiliated Hospital of Soochow University, Suzhou 215123, China

**Keywords:** ionizing radiation, polydatin, intestinal radiation damage, amniotic fluid stem cells, exosomes

## Abstract

This study aimed to develop an exosome-coated polydatin (PD) nanoparticles (exo-PD) for improving the water solubility and bioavailability of polydatin and explore its salutary effects on intestinal radiation injury. Exosomes (exo) were extracted from the medium of human amniotic fluid stem cells (hAFSc). Mice were divided into control group, irradiation (IR) group, irradiation+PD (IR+PD) group, irradiation+exo (IR+exo) group and irradiation+exo-PD (IR+exo-PD) group. The results of characterization of protein markers, particle size, morphology and cellular uptake ability confirmed that exosomes were effectively isolated using ultracentrifugation. Compared with the IR group, exo-PD improved cell viability, prolonged survival of mice, improved leukocyte count and reduced diarrhea rate. Histological results showed that the exo-PD group had significant improvements in small intestinal villus length and crypt number and less crypt cell damage. exo-PD could reduce IL-1α and IL-6 levels, reduced γ-H2AX expression, increased mitochondrial membrane potential, enhanced oxidative phosphorylation, and delayed cellular senescence. exo-PD could alleviate intestinal injury by improving mitochondrial function through PI3K-AKT pathway. The exo-PD was able to reduce radiation damage to intestinal cells and could be a potential candidate for salvage of intestinal radiation damage.

## INTRODUCTION

The intestinal mucosa is a highly radiation-sensitive tissue [1]. In a nuclear emergency, the intestine can be severely damaged by radiation at high doses, and the outcome of the treatment can directly determine the outcome of the patient [2]. In about 50% of patients treated with radiation therapy for clinical abdominopelvic tumors, radiation damage to the bowel would occur as a bad effect [3]. In order to prevent and control intestinal radiation damage, researchers have done and found some effective compounds, such as sulfur-containing compounds, biogenic amines, estrogens and their derivatives, natural drugs (such as polysaccharides, coumarins, alkaloids, etc.) and cytokines [4, 5]. However, none of these compounds have been formally introduced into clinical use due to their side effects or unsatisfactory therapeutic effect [6]. Burdelya et al. reported in 2008 that the Salmonella flagellin CBLB502, which activates the NF-kappaB pathway via Toll-like receptor 5 (TLR5), was an excellent treatment effect for intestinal radiation injury in mice exposed to high doses of radiation [7]. However, the drug approved for clinical use is only Amifostine (WR-2721) [8]. There are no specific drugs approved for the treatment of intestinal radiation injury, and the main clinical solutions is on adjuvant supportive therapy, including anti-inflammatory or anti-infective treatment, and maintaining the balance of water and electrolyte [9]. Therefore, it is vital to find effective drugs to combat radiation damage to the intestine.

In a previous study, our team explored polydatin (PD) as a therapeutic agent for intestinal radiation injury [10]. However, its poor water solubility makes it difficult to administer orally, while intravenous and abdominal administration are also difficult to reach the sites of injury in the intestine with targeted enrichment. Therefore, we attempted to find an ideal drug carrier that could assist to carry PD in intraperitoneal administration and make them targetable, increasing the biocompatibility and tissue availability.

Exosomes are 30–100 nm membrane-like vesicles secreted by a variety of cells, especially stem cells, containing a variety of DNA, RNA and abundant transmembrane proteins, have attracted extensive basic science and clinical research about its physiological and histological effect [11–14]. Meanwhile, as an excellent vesicular nanoparticle, it is a good carrier for small molecule drugs and has been increasingly used in biomedical engineering in recent years [15]. In previous studies in our laboratory, it was demonstrated that using amniotic fluid stem cells can be differentiated into keratin-forming cells and sweat-gland forming cells and can be used in a low immune microenvironment for skin wound repair [16, 17]. Stem cells can produce large numbers of exosomes compared to other cells, and many regenerative properties previously attributed to stem cells have been shown to be mediated by paracrine production of exosomes [18]. Wiklander et al. [19] noted that exosomes are usually distributed to organs of the mononuclear phagocyte system, with the highest accumulation in the liver, followed by the spleen, gastrointestinal tract and lung. Based on these properties of stem cell-derived exosome [20], we intended to use exosomes extracted from amniotic fluid stem cells as a targeting vehicle for PD to treat the intestinal damage of radiation [21].

In this study, we extracted amniotic fluid stem cell exosomes, synthesized exo-PD nanomedicines, observed the prevention and treatment effects of novel nanodrug on intestinal radiation injury, and explored the molecular mechanisms. Exosome is an excellent natural carrier, making the encapsulated exo-PD highly bioavailable, soluble and safe [22], and reaching higher concentration in the gastrointestinal tract. Thus, nanoparticles formed by encapsulation of PD using exosomes will be an excellent radioprotective nanodrug.

## MATERIALS AND METHODS

### Animals

6–8-week-old SPF-grade C57BL/6J mice, whose mean weight is (25.78 ± 0.94) g, were purchased from Vital River (China). After that, they were housed in the barrier system of Soochow University Laboratory Animal Centre and operated according to the relevant SPF regulations. During the survival tests, the mice were observed and weighed daily before being returned to their cages. All handling procedures of animals were reviewed and approved by the Animal Ethics Committee of Soochow University.

### Reagents

HIEC-6 cells were purchased from ATCC (USA) and the medium, fetal bovine serum and trypsin used were purchased from Gibco. PD was purchased from Meilun Biological Company (China) and Seahorse XF related reagents were purchased from Agilent (USA). Did staining solution, JC-1 staining solution and β-galactosidase staining kit were purchased from Beyotime (China). UV-Vis NIR spectrophotometer was from Shimadzu Corporation (Japan). Transmission Electron Microscope (TEM, TecnaiG20) was from FEI (USA). Biological X-ray Irradiator (X-RAD320IX) was from PXi Corporation (USA).

The list of antibodies used is as follows: p-PI3K: Cell Signaling (17366s); PI3K: Cell Signaling (4263s); p-AKT: Cell Signaling (4058s); AKT: Cell Signaling (9272s); γ-H2AX: Cell Signaling (25775); NF-kappaB: Epitomics (2229-1); IL-1 Ra: Abcam (ab124962); IL-6: Abcam (ab259341); β-actin: Abcam (ab133626). The rest of the reagents were maintained and commonly available in the laboratory.

### hAFSc culture and freeze storage

The hAFSc was donated by the Institute of Stem Cell Research, Soochow University. The frozen hAFSc was thawed from the liquid nitrogen tank and centrifuged, then passed into three 10 cm diameter cell-culture dishes at a ratio of 1:3 with the addition of antibodies. Dishes with 8 ml of full medium (FUJIFILM Irvine Scientific, 99473) were placed in a 5% CO_2_ incubator and waited for the cells to adhere to the wall. Amniotic fluid stem cells can be considered for passaging when they reach 80% of maximum growth density. Cells between the P3 and P8 generations were selected for the experiment.

### Exosome extraction

The gold standard for exosome isolation is briefly summarized as centrifuging the collected P3 and P8 generations of well grown amniotic fluid stem cell in 50 ml centrifuge tubes at room temperature for 10 min at 300 g, 10 min at 2000 g and 30 min at 10000 g. Each time the supernatant is collected and centrifuged again to remove live cells, dead cells, cell debris and large vesicles in turn. Finally, the supernatant of last step centrifuge at 4°C for 70 min at 120000 g to obtain exosomes from the bottom of the tube. The exosome solution is obtained by re-centrifugation with PBS and storage in a refrigerator at −80°C after filtering with a 0.22 μm filter.

### Western blot

This experiment was divided into the assay of exosome marker proteins and the assay of mouse intestine-associated proteins. Total exosome protein is extracted according to the method in the literature by taking an appropriate amount of exosome suspension and lysing it directly 1:1 with tissue protein lysis solution on ice for 30 min to release the protein molecules from the exosomes and then boiling it with loading buffer for protein imprinting. For the detection of mouse intestinal tissue-associated proteins, the small intestinal proteins of all groups were taken and lysed. After BCA proteins were quantified, samples were subjected to SDS-polyacrylamide gel electrophoresis. Finally, the membrane that carried proteins was incubated with antibodies and exposed for imaging.

### Observation of exosome morphology by transmission electron microscopy (TEM)

At room temperature, 10 μl of the extracted exosome sample was added dropwise to the TEM copper grids and left for 1 min before blotting up the solution with filter paper. A further 10 μL of 3% phosphotungstic acid solution was added dropwise to each copper mesh for negatively staining for 1 min before blotting up. The copper grids were placed in a six-well plate that was put overnight in an oven at 70°C. The morphology of the exosomes after negative staining was observed by TEM and images were taken.

### Preparation of exosome-coated polydatin (exo-PD) nanodrug

Firstly, 1 mg/mL of PD standard solution was prepared in a volumetric flask and stored in a sealed container at 4°C away from light. After protein concentration of extracted exosome was measured, the exosome solution was diluted to 1 mg/mL with PBS. 990 μL of exosome solution were mixed with 10 μL of PD standard solution to obtain a new solution with an exosome concentration of 0.99 mg/mL and PD concentration of 10 μg/mL by repeated ultrasound and freeze-thaw treatment. Briefly, it was thawed at room temperature and the freeze-thawing process was repeated 3 times to obtain the exo-PD solution.

### Culture and viability assay of intestinal epithelial cells

Human normal small intestine cell line (HIEC-6) was cultured by using Opti-MEM (1×) Reduced Serum Medium (containing 4% fetal bovine serum, 20 mM Hepes, 10 mM Glutamax, 10 ng/ml EGF) in an incubator set at 37°C with saturated humidity and 5% CO_2_. When the HIEC-6 cells grew to about 80% density, cells were counted and passed into 96-well plates with 5000 cells per well, divided into 8 groups: IR group, 50 μg/ml group, 75 μg/ml group, 100 μg/ml group, 125 μg/ml group, 150 μg/ml group, 175 μg/ml group and 200 μg/ml group. The 96-well plates would be irradiated with a total dose of 8 Gy at 1 Gy/min under a Bio-X-ray irradiator. After irradiation, the cell medium would be exchanged and the exosome solution was added to each group. After 24 h and 48 h of incubation, the cell viability of the IR group, IR+PD group, IR+exo group and IR+exo-PD group were then measured with CCK-8.

### Cellular uptake capacity test of exosomes

The exosome solution was incubated with 10 μM Did staining solution for more than 30 min at room temperature. The solution was then added to 100 kDa ultrafiltration tubes and centrifuged at 4000 g for 10 min. The remaining concentrate was rediluted to the original volume, filtered through a 0.2 μm membrane and stored at 4°C. Sterile round cell crawls were placed in the bottom of the wells of a 24-well plate. Then adding 1 ml of growth medium, 5000 HIEC-6 cells were incubated in 24-well plate overnight at 37°C in 5% CO_2_. After the cells were adhered, each well was added to 100 μl Did-labelled exosome and incubated for more than 1 h at 37°C, 5% CO_2_ environment. Cells were then washed and fixed before being stained by Hoechst cell nuclear staining solution at room temperature. The crawls were covered with anti-fluorescence quenching agent, sealed with nail polish, and stored away from light until the assay was performed.

### Mouse irradiation and drug administration

The mice were irradiated with a single dose of 16 Gy at a rate of 150 cGy/min. After irradiation, they were administered intraperitoneally with different drugs immediately, including PD solution at 20 mg/kg and exosome solution at 2 mg/kg. After administration, the mice were kept in a staging room for 4 d. After 4 d, blood was taken from the eyes of mice which would be euthanized after that, and tissue samples were collected. The blood was centrifuged at 4°C for 10 minutes at 3000 rpm to collect the supernatant which would be stored at −80°C.

### Open field test (OFT)

A black, uncovered OFT-100 chamber (52.5 cm × 52.5 cm × 41.5 cm), was set up with a curtain covering the top and white paper covering the bottom to keep the room closed and quiet. Each mouse was placed in the central compartment at the bottom of the box, and tester stayed 1 m away from the mouse to observe the computer screen and record its activity for 10 min using the camera to investigate the behavioral changes of the mice between the IR group and the IR+exo-PD group. At the end of each experiment, the box was cleaned of residues such as feces and urine. And the device and box were sprayed with 75% alcohol to remove the odor and foreign matter, and waited for five minutes to prevent interference with the other mice.

### Mouse tissue organ sampling and HE staining

Organs were taken from the mice and gently put in 4% paraformaldehyde for 8 hours immersing. Then they were stored in 70% alcohol for long-term storage. Subsequently, steps were followed by dehydration, transparency, embedding, sectioning and staining, before drying and sealing the sections. Tissue sections were analyzed by light microscopy (200×). The intestinal villi and intestinal crypts of mice for each group were measured. And a minimum of 20 intestinal villi and at least 10 intestinal tubes were necessary for testing in each group.

### Proteomics and phosphorylated proteomics analysis

HIEC-6 cells were inoculated in six-well plate, 10000 cells for each well and divided into IR group and IR+ exo-PD group. After 24 hours of 8 Gy irradiation, the cells were collected and frozen in liquid nitrogen immediately. The samples were sent to Jingjie Biological Company (China). The detection and analysis of proteomics and protein phosphorylation were assisted by Jingjie Biological Company. Then expression and interaction of differential protein would be analyzed according to the results of mass spectrometry.

### Mitochondrial stress test

Testing was carried out using the Seahorse XFe24 instrument according to the instructions. The test is divided into pre-testing, which involves pre-warming the system, inoculating walled cells, cell irradiation treatment and hydrating the probe plate. The latter includes preparing the seahorse assay solution, washing the cells, preparing the working solution and loading the sample, before running the instrument to collect the data.

### Mitochondrial membrane potential assay experiment

HIEC-6 cells were planted in six-well plate and divided into four groups: IR group, IR+PD group, IR+exo group and IR+exo-PD group. 12 h later, the plate was irradiated with 8 Gy at a dose rate of 1 Gy/min. After irradiation, 100 μL of exo-PD solution were added to the IR+exo-PD group, 100 μL of exosome solution were added to the IR+exo group, 100 μL of PD solution were added to the IR+PD group and an equal amount of PBS was added to the IR group, and all groups were incubated for 24 h at 37°C, 5% CO_2_. JC-1 staining buffer (1×) was prepared according to the instructions. After incubation, fixation and washing, round cell crawls were removed with forceps, sealed by oil and examined quickly using a confocal microscope.

### Cellular senescence assay

We used β-galactosidase staining to detect cellular aging. After irradiation, 100 μl of different kinds of solution were added to each relating group respectively and all groups were incubated for 24 h. After that, the medium was aspirated from each well and washed once with PBS. 1 ml of staining fixative was added to fix the cells, which were then aspirated and washed with PBS after 15 min. Then 1 ml staining working solution was added to the six-well plate for hatching overnight at 37°C in a CO_2_-free incubator. The result would be observed and captured under a fluorescent microscope.

### Statistical processing

The images and data were processed and analyzed using GraphPad Prism and Image Pro Plus and SPSS software. Differences between two groups of data and statistical significance were analyzed by Student’s *t*-test or one-way analysis of variance (ANOVA). Survival curves were generated using the Kaplan-Meier method, and the differences were assessed by a log-rank test. ^*^ mean as *P* < 0.05, and ^**^ as *P* < 0.01, and ^***^ as *P* < 0.001.

## RESULTS

### Preparation and characterization of human amniotic fluid stem cell exosomes

**Figure 1:**
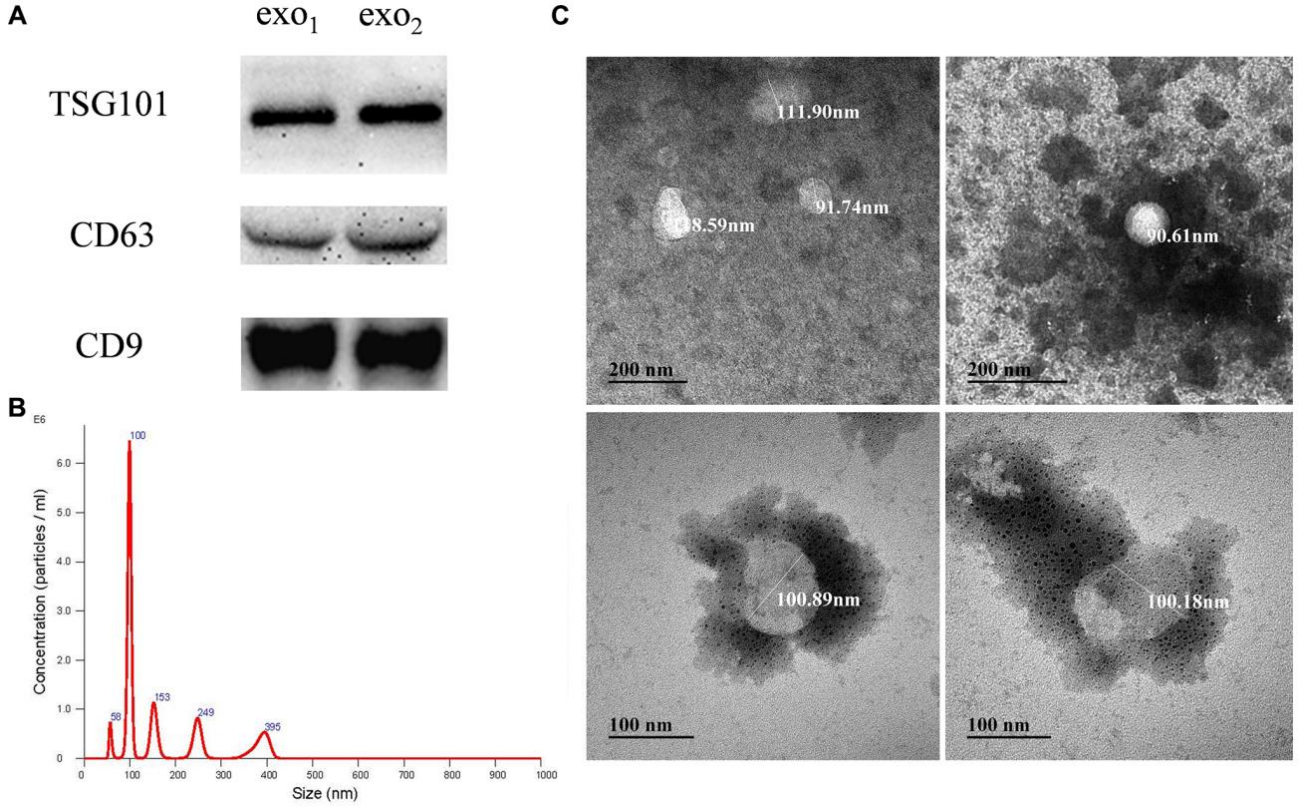
**Isolation and identification of human amniotic fluid stem cell exosomes.** (**A**) Expression levels of the exosomal signature expression proteins: TSG101, CD63 and CD9. exo_1_ and exo_2_ represent exosomes extracted from two parallel experiments; (**B**) Distribution of exosomal NTA particle size; (**C**) Morphology of the exosomes under transmission electron microscopy.

The P3 and P8 generations of hAFSc were selected, when the cells were in the best state of growth. Exosomes from amniotic fluid stem cells were extracted using ultracentrifugation and characterized. As shown in Figure 1A, Western blot detected positive expression of the exosome marker TSG101 and transmembrane proteins CD63 and CD9 in the samples, which were consistent with the characteristics of exosome protein markers. As shown in Figure 1B and 1C, the NTA results showed that the particle size of the extracted exosome was concentrated around 100 nm, while the vesicles in the transmission electron microscopic field showed a spherical shape with membrane structure, which proved that the extracted exosome had the structural characteristics of phospholipid bilayer. The average particle size of the extracted exosomes was found to be (106.30 ± 11.19) nm, which was consistent with the reported particle size of exosomes of 30–150 nm. Combined with the above characterization, it proved that the exosome from amniotic fluid stem cells was successfully extracted and could be used for subsequent experiments.

On the other hand, the exosomes we extracted were stored in a refrigerator at 80°C. After repeated freezing and thawing, exosomes inevitably condense into clumps (As shown in the Figure 1A, 2B below). So, after PD was encapsulated by exosomes, the morphology of exosome under electron microscopy may not accurate. At the same time, multiple protein concentration measurements of exosomes indicated that the concentration of exosomes is very low, so the concentration of PD in the package is certainly not high (As shown in the Figure 1C). We tried to measure the concentration of the PD wrapped in exosome at 306 absorption spectrum, the average concentration of exo-PD is between 10–20 ng/ul. Therefore, industrial methods are still needed to improve the concentration.

**Figure 2:**
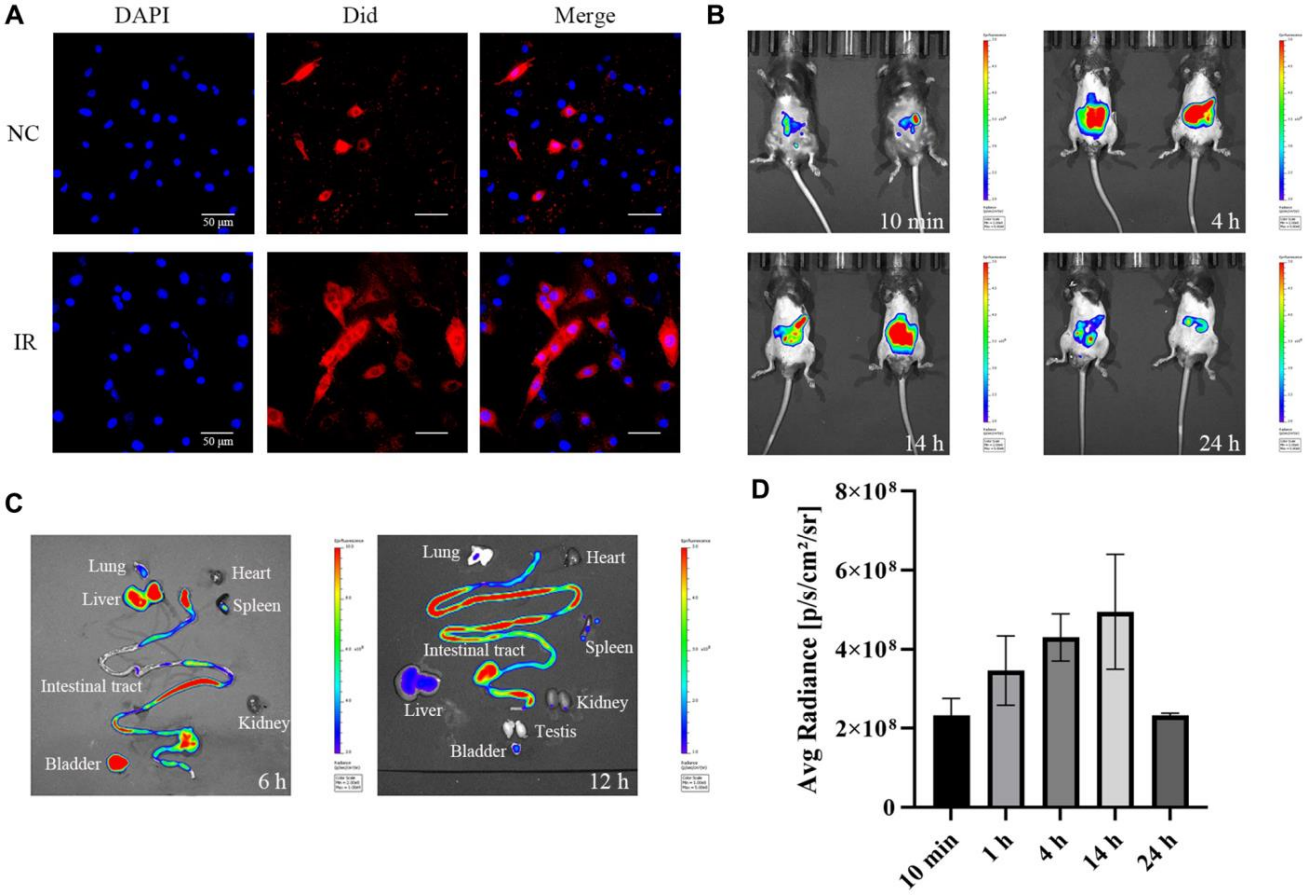
**Cellular uptake of exosomes *in vitro* and metabolism of exosome *in vivo*.** (**A**) Uptake of exosomes by HEIC-6 cells and intraperitoneal metabolism of exo-ICG. (**B**) Fluorescence values of the abdomen of mice injected intraperitoneally with exo-ICG at 10 min, 4 h, 14 h and 24 h. (**C**) Fluorescence values of the abdominal organs of mice at 6 h and 12 h. (**D**) Fluorescence quantification values.

### Cellular uptake of exosomes and *in vivo* metabolism in mice

Did dye was loaded into purified exosomes using the repeated freeze-thaw method and then the Did-loaded exosome was added to HIEC-6 cell culture media of unirradiated group (NC) and post-irradiated group. As shown in Figure 2A, the distribution of red fluorescent markers of exosomes appears extracellularly and intracellularly, which indicates that the intestinal epithelial cells can take up exogenously added exosomes. The ability of the cells to take up exosomes was significantly enhanced after IR. The cellular uptake of large amounts of exosomes resulted in the expansion of Did dye throughout the cytoplasm and cell membrane. This indicates that the isolated exosomes were still biologically active. exo-ICG (exosome incubated with indocyanine green (ICG)) was then used to simulate the release and uptake of exo-PD in the intestinal lumen and the results are shown in Figure 2B and 2D. After intraperitoneal injection (i.p.) of exo-ICG, a strong signal could be rapidly detected in the abdominal cavity of mice. The abdominal signal gradually increased with time, and the signal started to decrease after about 14 h. Figure 2C shows that there were strong fluorescence values in the liver, small intestine and bladder of mice after 6 h, reflecting the enrichment of exo-PD in the organs after intraperitoneal injection. 12 h later, the fluorescence values of all organs in the abdominal cavity started to decrease, but there were still strong fluorescence values in the small intestine, indicating that exo-PD had a longer duration of action in the small intestine.

### Radioprotection of exo-PD on small intestinal epithelial cells

**Figure 3:**
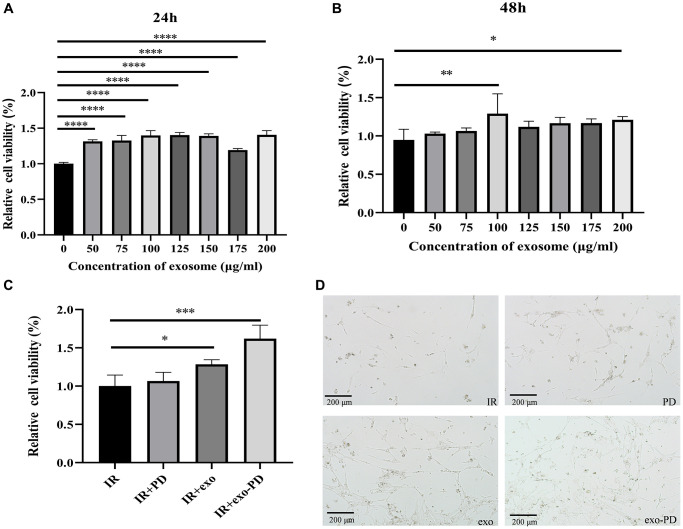
**CCK-8 assay of small intestinal epithelial cells after exosome and exo-PD treatment.** (**A**) Cell viability of intestinal epithelial cells after IR at different exosome concentrations after 24 h treatment. (**B**) Cell viability of intestinal epithelial cells after IR at different exosome concentrations after 48 h treatment. (**C**) Cell viability of different groups after IR and treating with PD (10 μg/ml), exosome (exo, 100 μg/ml) and exo-PD (10 μg/ml) for 24 hours respectively. (**D**) Cell status observed microscopically after 72 h. Statistical plots with ^*^ as *p* < 0.05, ^**^ as *p* < 0.01 and ^***^ as *p* < 0.001 indicate that the differences between groups are statistically significant.

The CCK-8 assay was used to detect the effect of amniotic fluid stem cell exosome on intestinal epithelial cells. Figure 3A and 3B show that the addition of only amniotic fluid stem exosomes after IR resulted in a significant increase in cell viability no matter 24 h or 48 h later. After 24 h, each group had a statistically significant *p*-value of less than 0.0001 compared to IR alone. After 48 h, cell viability was also elevated in each group at exosome concentrations of more than 100 μg/ml compared to the IR group (*p* < 0.05). As shown in Figure 3C and 3D, after 24 h, the cell viability after IR treated with exo-PD was significantly higher than that of the IR group. And after 72 h, most of the cells in the IR group peeled off the culture dish and very few cells survived. In contrast, with the addition of exosome and exo-PD, some cells were still adhered to the bottom of the culture dish and could still passage to survive. The experiments suggest that the extracted amniotic fluid stem exosomes and the synthesized exo-PD have therapeutic effects on the radiation damage of small intestinal epithelial cells *in vitro*. For the follow-up experiments, 100 μg/ml of exosomes was used for PD incubation and 24 h was chosen as the time point for cell survival observation.

### Rescue effect of exo-PD on intestinal radiation injury in mice

**Figure 4:**
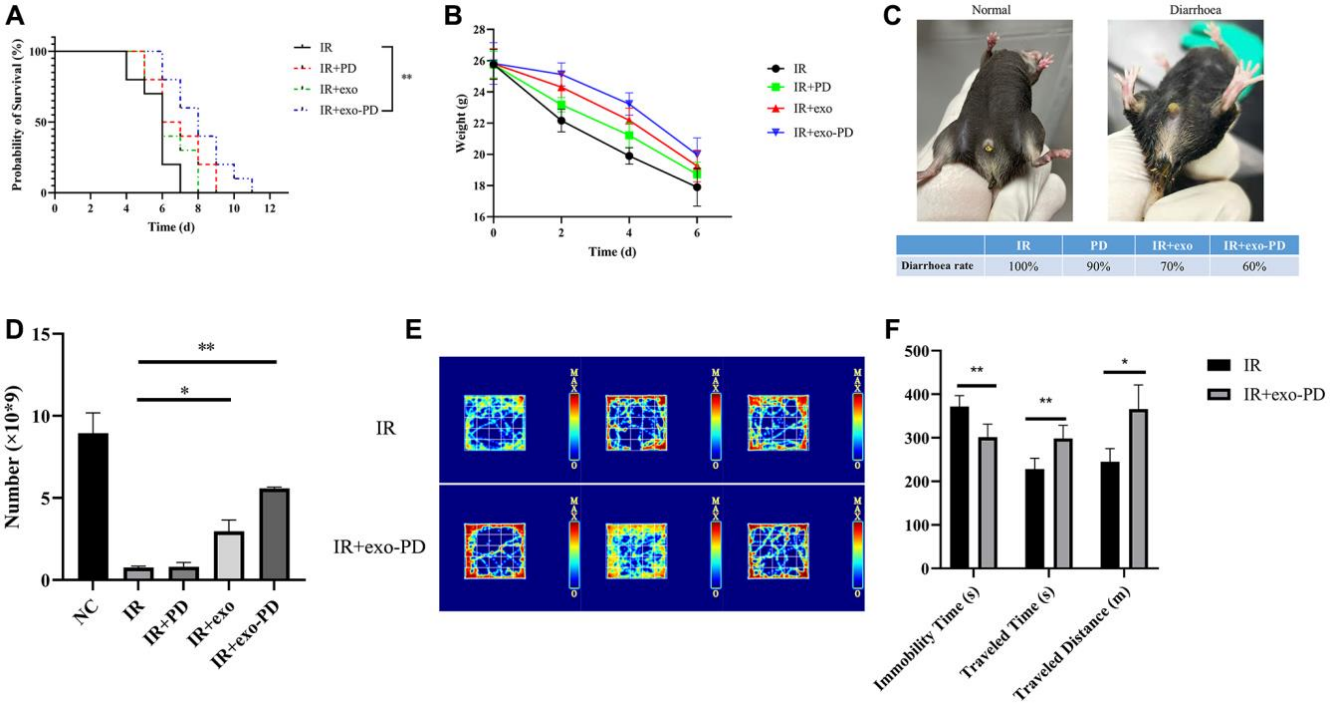
**Effect of PD, exo and exo-PD on survival, body weight, diarrhea rate, leukocyte change and behavior of mice after irradiation.** (**A**) Effect of exo-PD on survival of mice after IR. (**B**, **C**) Differences in body weight changes and diarrhea rate of mice in different groups after IR. (**D**) Leukocyte changes on day 4 after irradiation in mice from different treatment groups. (**E**, **F**) Heat map and statistical results of the open field experiment in mice from two treatment groups, and black represents mice in IR group and gray represents mice in exo-PD group. PD solution, exosome solution and exo-PD solution were all injected intraperitoneally immediately after irradiation within half an hour. Data are presented as mean $\bar{X}\pm \text{SEM},$* n* = 10. Statistical plots with ^*^ as *p* < 0.05, ^**^ as *p* < 0.01 and ^***^ as *p* < 0.001 indicate statistically significant differences between groups.

Male mice at 6–8 weeks were divided into four groups (10 mice each) and given a lethal dose of 16 Gy (dose rate 1.5 Gy/min) of abdominal irradiation. Then they were given different treatments to observe survival times. Figure 4A shows that the mean survival time in the IR group was 5.7 d, with a median survival time of 6 d. While the mean survival time in the exo-PD group was 8.1 d, with a median survival time of 8 d (*p* = 0.001). Also, changes of body weight, diarrhea, leukocyte and behavioral in the mice due to radiation were recorded. As shown in Figure 4B, mice in the IR group had the fastest weight loss, from a mean of (25.76 ± 0. 86) g to (17.9 ± 1.08) g, 30.51% loss in 6 days. In contrast, the exo-PD group lost 22.62% of their body weight from (25.82 ± 1. 19) g to (19.98 ± 0. 97) g in 6 days. As shown in Figure 4C, without intervention, all the irradiated mice would have developed diarrhea on the fourth day. In contrast, PD group, the exo group and the exo-PD group could reduce the diarrhea rate appropriately. Among them, the exo-PD group could significantly alleviate the diarrhea in mice. Since most of the mice would die on day 6, the weight changes had been recorded until the end of day 6. Figure 4D shows that the normal mouse leukocyte count was (8.49 ± 1.24) × 10^9^, which decreased to (0.755 ± 0.092) × 10^9^ on the fourth day after irradiation, while the exo-PD group was (5.58 ± 0.071) × 10^9^. In order to observe the behavioral changes in mice after irradiation and to show the activity and stress response of mice after irradiation, the open field test in the IR group and IR+exo-PD group had done. Figure 4E and 4F show that mice in the IR+exo-PD group showed a significant increase in horizontal movement time (*p* < 0.01) and distance (*p* < 0.05) and a significant decrease in central compartment dwell time (*p* < 0.001) compared to mice in IR group, indicating that mice given the IR+exo-PD group were more active and had an increased desire to explore.

**Figure 5:**
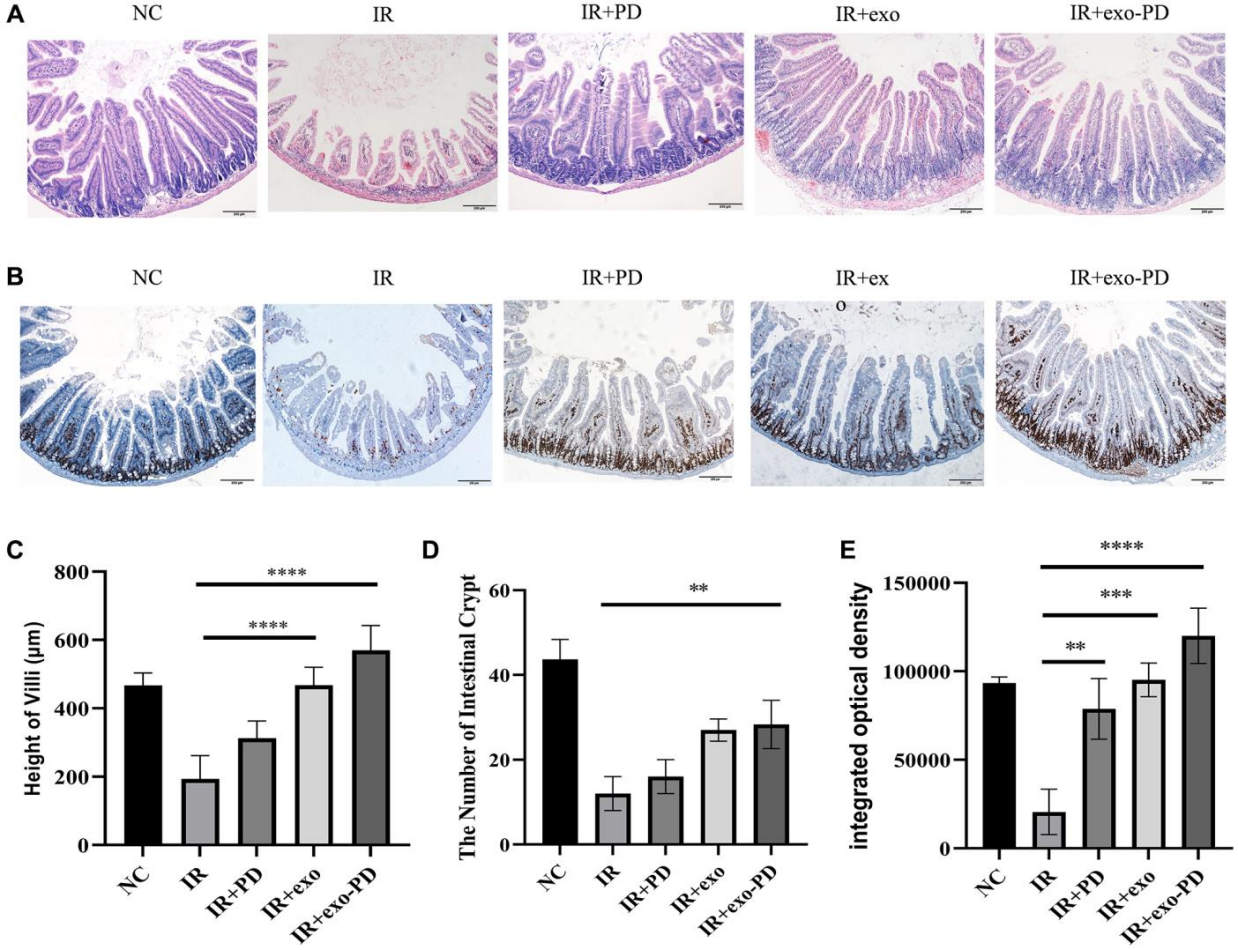
**Small intestinal HE and immunohistochemical results after IR in mice.** (**A**) Intestinal HE for each group of mice after irradiation. (**B**) Ki67 staining results of intestinal tissue. (**C**, **D**) Statistical plots of height of villi and number of intestinal crypts reflecting the results of Figure (**A**). (**E**) Integrated optical density about Ki67 staining results of intestinal tissue from Figure (**B**). Three typical sections for each group, comparisons between 100× and 200×, 4 fields of view per section. Statistical plots with ^*^ as *p* < 0.05, ^**^ as *P* < 0.01, ^***^ as *p* < 0.001 and ^****^ as *p* < 0.0001, indicating statistically significant differences between groups.

We conducted another abdominal irradiation at 16 Gy and took the intestine canals of irradiated mice on day 4 to count the length of intestinal villi and the number of intestinal crypts. As showed in Figure 5A, 5C and 5D, the intestinal villi of the irradiated mice were mostly shed and the length of the intestinal villi and the number of intestinal crypts were significantly reduced in the IR group. The number of intestinal villi and crypt was significantly increased when giving exosomes (exo) and exo-PD in mice after irradiation, showing that both exo and exo-PD had protective effect, of which the latter performed better.

Intestinal stem cells are located at the base of the intestinal crypts and play an important role in the stabilization and renewal of the intestinal epithelial lining. Ki67 immunohistochemistry was performed on intestinal tissues. As shown in Figure 5B and 5E, the integrated optical density (IOD) values of Ki67^+^ cells in the IR group were significantly lower than those in the NC, IR+PD, IR+exo and IR+exo-PD groups. Consistent with the HE results, the IR group had low crypt numbers and abnormal morphology, and intestinal cell renewal was impaired, while PD, exo, and exo-PD could protect the mouse intestine and promote the recovery of intestinal cells.

### Screening for differentially expressed proteins in intestinal villi and crypts after irradiation in mice

**Figure 6:**
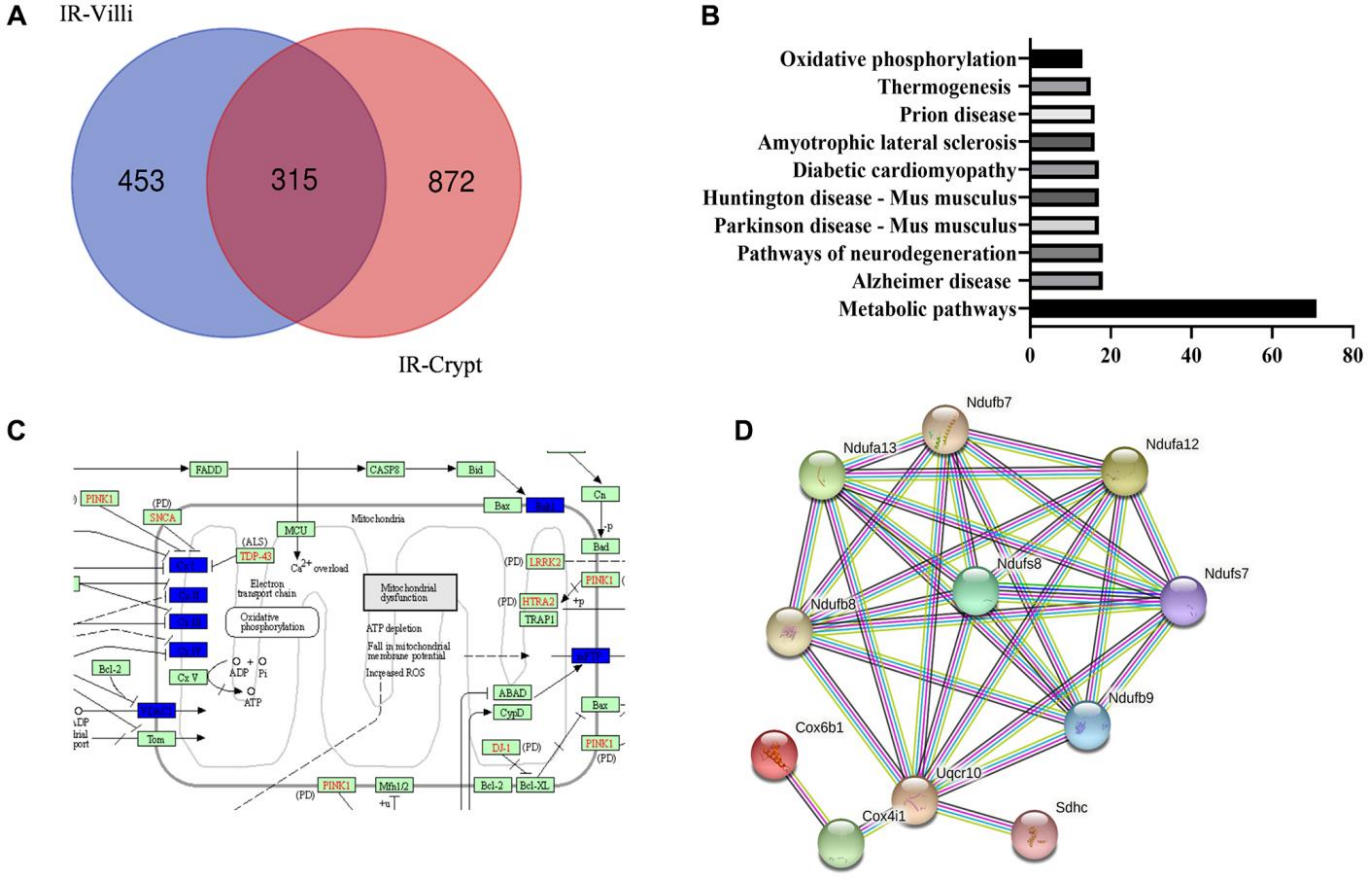
**Common differential genes of intestinal villi and intestinal crypts after IR from the result of protein mass spectrometry.** (**A**) Venn diagrams. (**B**) GO enrichment of the identified common differential genes. (**C**) KEGG mapper on mitochondrial oxidative phosphorylation-related pathways. (**D**) STING on the few proteins with the highest interactions.

Since the protein spectra of intestinal villi and crypts of mice before and after irradiation were made in the previous laboratory, we had obtained the differential proteins of intestinal villi and crypts of mice 6 h after irradiation. So we made Venn diagrams of the differential proteins to find out the proteins that were jointly reduced and increased by intestinal villi and crypts. As shown in Figure 6A and 6B, intestinal villi were screened for a total of 315 common differential proteins. GO enrichment analysis of the common proteins revealed that they were mostly enriched in various metabolic pathways.

We then further performed KEGG analysis of metabolic proteins. As shown in Figure 6C and 6D, we found that the common difference proteins were more concentrated in mitochondrial oxidative phosphorylation-related proteins and had a high degree of STING interactions (>90%). We then hypothesized whether exo-PD could alleviate intestinal radiation damage by improving mitochondrial function.

### exo-PD ameliorated intestinal epithelial cell senescence and death by alleviating mitochondrial damage

**Figure 7:**
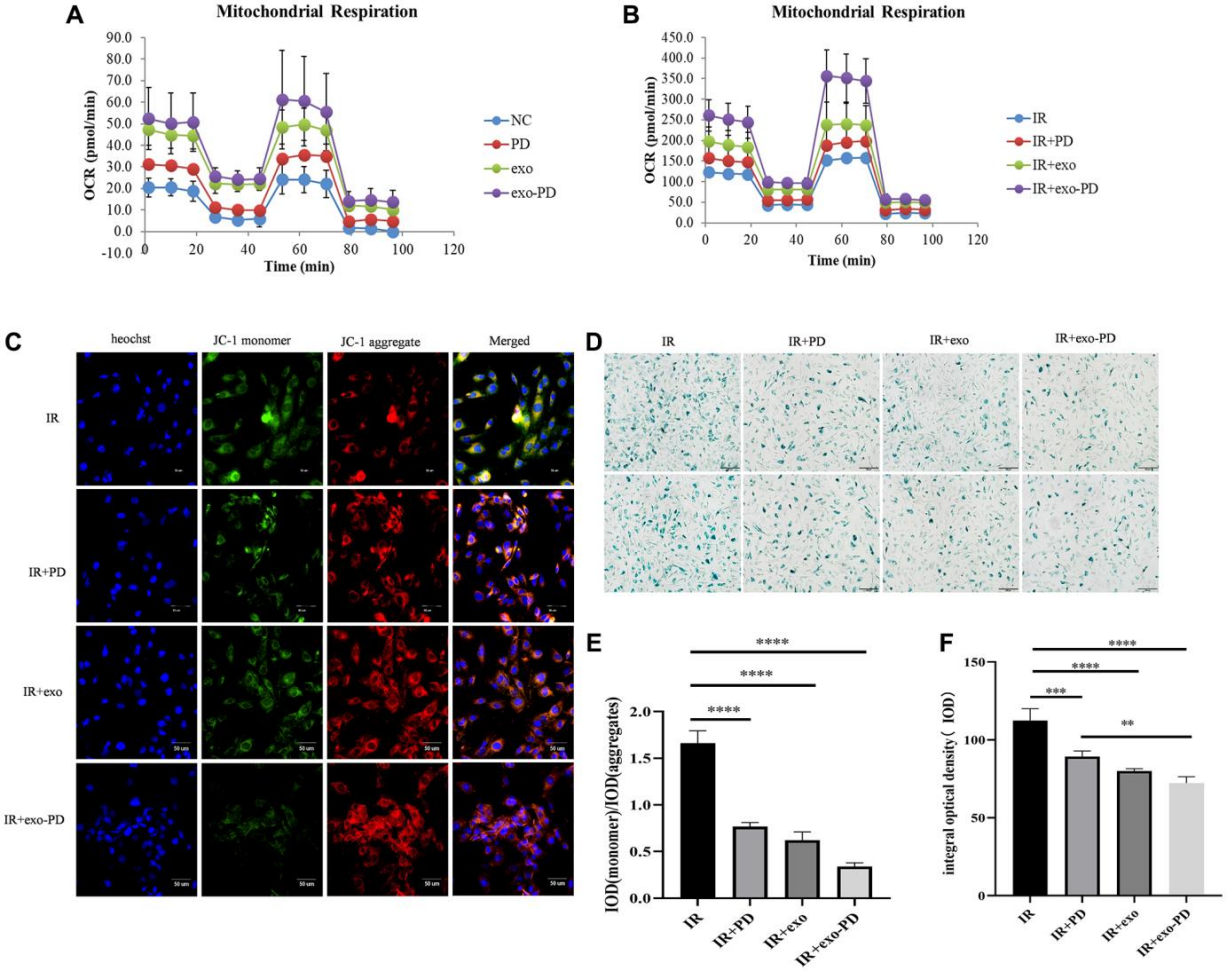
**exo-PD regulates mitochondrial oxidative phosphorylation pressure, mitochondrial membrane potential and cellular senescence state in intestinal epithelial cells.** (**A**) Mitochondrial oxidative respiratory pressure test. (**B**) Oxidative respiratory pressure of HIEC-6 after 8 Gy irradiation and 24 h incubation with the addition of PBS, PD solution, exosome solution and exo-PD solution. (**C**) exo-PD ameliorates the decrease in mitochondrial membrane potential of intestinal epithelial cells. (**D**) exo-PD retarded the senescence of intestinal epithelial cells. (**E**) The ratio of IOD between monomer and aggregates about mitochondrial membrane potential. (**F**) The IOD of β-galactosidase staining of intestinal epithelial cells in IR group, IR+PD group and IR+exo-PD group. ^*^ represents *p* < 0.05, ^**^ represents *p* < 0.01, ^***^ represents *p* < 0.001, and ^****^ is *p* < 0.0001, indicating that the differences are statistically significant.

Mitochondria are the center of cellular energy metabolism and play an important role in the regulation of cellular metabolism, signal transduction and ROS catabolism [23]. In particular, mitochondrial homeostasis can prevent apoptosis or necrosis when cells are subjected to high external stress [24]. We chose the seahorse mitochondrial respiratory stress test to observe mitochondrial metabolism. As shown in Figure 7A and 7B, the basal respiration, ATP production and oxidative respiration maximal potential of cells in the exo-PD group were better than those in other groups. This shows that exo-PD improved the mitochondrial oxidative respiration capacity and enhanced the potential of mitochondrial oxidative phosphorylation.

The decrease of mitochondrial membrane potential is a hallmark event in the negative environment of cellular senescence, early apoptosis and autophagy [25]. The decline of cell oxidative phosphorylation after irradiation may be related to the imbalance in the decrease of cellular mitochondrial membrane potential due to irradiation. The decrease in mitochondrial membrane potential can be easily detected by the shift in red and green fluorescence of cell mitochondria after JC-1 staining and the IOD value after merge can also be used as a detection indicator for negative cellular events. As shown in Figure 7C and 7E, the exo-PD nanoparticles resulted in more JC-1 molecular polymers and fewer monomers in small intestinal epithelial cells after IR, and its ratio of IOD_green_/IOD_red_ was statistically significant compared to ratio of the IR group.

Ionizing radiation is one of the causes of mitochondrial damage, which leads to stem cell senescence and death [26–28], thus impairing stem cell self-renewal and differentiation [29]. As shown in Figure 7D and 7F, PD, exo and exo-PD reduced cellular senescence caused by irradiation and normalized the rate of intestinal cell renewal compared to the IR group, consistent with the results of the previous experiments that increased cell viability.

### exo-PD improves cellular mitochondrial oxidative respiration via the PI3K-AKT pathway

**Figure 8:**
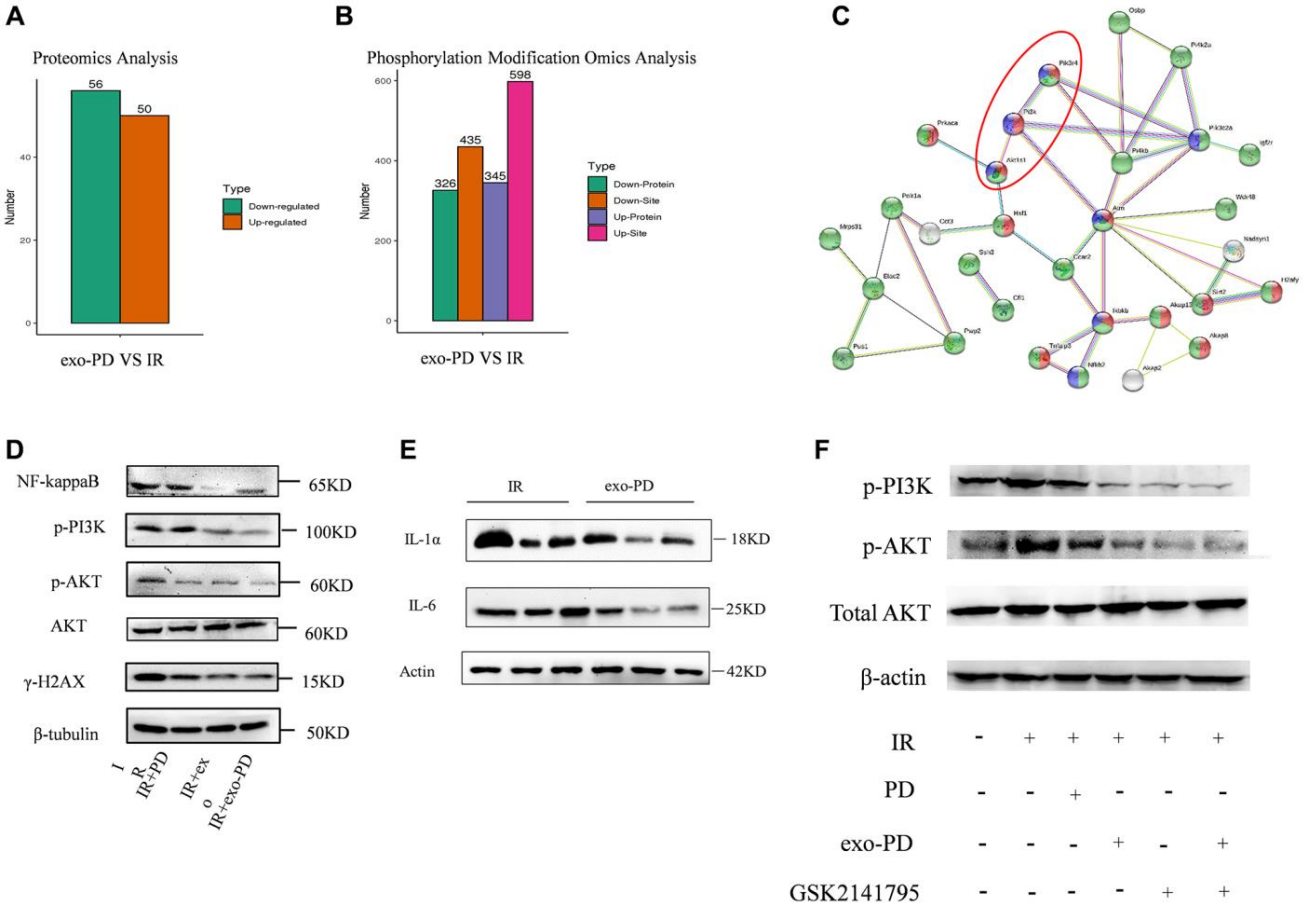
**exo-PD acts on intestinal cells via the PI3K-AKT pathway as determined by phosphorylated protein profiling.** (**A**, **B**) Differential proteins and differential loci derived from proteomic and phosphorylated proteomic modification assays. (**C**) STING protein interactions. Red represents phosphorylation regulation, blue represents PI3K-AKT signalling pathway and green represents intracellular membrane-bound organelles. (**D**) Effect of PD, exo and ex-PD on protein expression of PI3K/AKT and γ-H2AX in mouse intestine after 24 h of IR. (**E**) Western blot of IL-1α and IL-6 in mouse intestine after exo-PD treatment. (**F**) Expression of PI3K and p-AKT after treatment with different conditions following the addition of AKT inhibitors.

In order to find specific targets of exo-PD affecting mitochondria, we considered that mitochondrial oxidative respiratory proteins may be related to molecular phosphorylation. We subsequently collected HIECs with treating of 8 Gy irradiation and exo-PD incubation. Proteomics and phosphorylated proteomic modifications were examined. As shown in Figure 8A and 8B, 106 differential proteins, 671 phosphorylated differential proteins and 1033 phosphorylated differential sites were obtained. We then identified mitochondrial oxidative respiration-related proteins and proceeded to do STING protein interactions, shown in Figure 8C. Several possible pathways were derived: PI3K-AKT signaling pathway, NF-kappaB pathway, AMPK pathway and CDK1-ATM-sirt2 pathway, etc. After long deliberation, we explored whether exo-PD improves mitochondrial function in intestinal cells and affects cellular status through the PI3K-AKT signaling pathway.

As shown in Figure 8D and 8E, radiation activated PI3K-AKT expression, while protein expression of p-AKT was lower in the IR+PD group, IR+exo group and IR+exo-PD group than the expression in the IR group, supporting the hypothesis that PD, exo and exo-PD improved mitochondrial function by regulating PI3K-AKT pathway. Meanwhile exo-PD was able to reduce the production of IL-6 and IL-1α and attenuate the inflammatory effect. The reduced expression of γ-H2AX also showed a reduction in DNA double-strand breaks.

We then chose the AKT inhibitor GSK2141795, a small molecule metabotropic inhibitor that is highly selective for AKT 1/2/3. As shown in Figure 8F, IR elevated the expression of PI3K and p-AKT in normal intestinal epithelial cells, while exo-PD and GSK2141795 had a common effect of reducing PI3K and p-AKT. And exo-PD was more effective than PD, demonstrating that exo-PD can reduce radiation damage in intestinal epithelial cells via PI3K-AKT.

## DISCUSSION

In recent years, exosome-derived nanodrugs have received increasing attention from scientists [30]. For example, exosomal adriamycin nanodrugs were successfully engineered from ginger-derived exosomes and were able to effectively internalize into and inhibit the growth of Colon 26, a type of colon cancer model cells [31]. Whereas current rescue drugs for radioactive intestinal injury focus on reducing DNA breakage, antioxidant production, scavenging free radicals and inhibiting lipid peroxidation [32–34]. Therefore, both hAFSc-derived exosomes and exosome-coated polydatin nanoparticles exo-PD have potential for therapeutic applications in intestinal radiation injury.

We prepared the polydatin-loaded exosome exo-PD using repeated freeze-thawing with ultrasound and further investigated the biological functions of exo-PD in *in vivo* and *in vitro* experiments. Exosomes can deliver lipids, proteins, RNA and DNA with or without direct contact with cells [35]. Therefore, as a carrier for polydatin, exo-PD may be superior to other synthetic nanoparticles. We found that exo-PD improved cell viability after irradiation and prolonged survival of mice. Meanwhile exo-PD could improve leukocyte count, delay the weight losing and reduce the rate of post-irradiation diarrhea. Histological results show that exo-PD increased the length of the villi and the number of crypts after IR, resulting in less damage to the crypt cells.

By analyzing the results of proteomic and phosphorylated proteomic from abdominally irradiated mice, we clarified that exo-PD alleviated radiation damage in intestinal cells by promoting the oxidative respiratory capacity of mitochondria. *In vitro* experiments showed that exo-PD slowed down the decrease of mitochondrial membrane potential after irradiation, which ensured basal respiratory capacity of cellular mitochondria. At the same time, the elevated mitochondrial activity attenuated radiation-induced senescence, maintaining a normal rate of intestinal cell renewal. Thus, exo-PD could improve mitochondrial oxidative respiratory capacity, keep mitochondrial oxidative phosphorylation potential and alleviate cellular radiation damage.

In this study, we screened PI3K-AKT pathway from results of proteomics and phosphorylated proteomics eventually, which increased mitochondrial membrane potential and enhanced oxidative phosphorylation to delay cellular senescence or death. exo-PD can also attenuate expressions of IL-1α and IL-6 in intestinal cells after radiation, as well as reducing γ-H2AX. This may be related to mitochondrial biogenesis [36]. Mitochondrial biogenesis is kind of cell physiological response to external stress, which may lead to increased energy demand. And it plays an important role in the regulation of cellular metabolism, signal transduction and mitochondrial ROS regulation [23, 37]. Mitochondrial biogenesis maintains cellular homeostasis by ensuring the quality of mtDNA and regulating organelle renewal. And it has been widely explored in recent years due to its relevance to human ageing, neurodegenerative diseases, cellular metabolic diseases, and tumours [38, 39].

The direct effect of IR on mitochondria (approximately 30% of total cell volume) leads to mitochondrial dysfunction, increases mitochondrial oxidative stress (MOS), and induces apoptosis [40]. IR induces intracellular and intercellular signaling in mitochondrial gene expression by regulating proteins involved in energy metabolism and antioxidant reactions, thus promoting cell survival [41, 42]. Therefore, the involvement of mitochondria in these reaction processes suggests that mitochondria play a crucial role in IR reactions. Recent studies have shown that scientists have shifted their research focus to using mitochondrial targets to treat cell survival and function. Potential mitochondrial targets providing cellular protection are those regulating permeability transition pores, electron transport chains (ETC), cardiolipin content, ion channels and transporters, ATP, mtDNA, and protein synthesis [43].

In summary, we successfully prepared the exosome-coated polydatin nanoparticles (exo-PD). exo-PD improved the mitochondrial oxidative respiration capacity of intestinal cells through inhibition of the PI3K-AKT pathway, reduced intestinal cell senescence and death, and improved rate and quality of survival in mice after high-dose irradiation. We have provided a new idea for traditional drugs to enter the clinic to rescue intestinal radiation injury.
